# InterferenceAnalyzer: Tools for the analysis and simulation of multi-locus genetic data

**DOI:** 10.1186/1471-2105-6-297

**Published:** 2005-12-12

**Authors:** Lalitha Viswanath, Elizabeth A Housworth

**Affiliations:** 1 School of Informatics, Indiana University, Bloomington, Indiana, 47405, USA; 2 Departments of Mathematics and Biology, Indiana University, Bloomington, Indiana, 47405, USA

## Abstract

**Background:**

Good statistical models for analyzing and simulating multilocus recombination data exist but are not accessible to many biologists because their use requires reasonably sophisticated mathematical and computational implementation. While some labs have direct access to statisticians or programmers competent to carry out such analyses, many labs do not. We have created a platform independent application with an easy-to-use graphical user interface that will carry out such analyses including the simulations needed to bootstrap confidence intervals for the parameters of interest. This software should make multi-locus techniques accessible to labs that previously relied on less powerful and potentially statistically confounded single interval or double interval techniques.

**Results:**

We introduce InterferenceAnalyzer, an implementation with a user-friendly graphical interface incorporating previously developed algorithms for the analysis and simulation of multilocus recombination data. We demonstrate the use and features of the program with an example of multilocus tetrad data from the mustard plant, *Arabidopsis thaliana*, and the yeast, *Saccharomyces cerevisiae*.

**Conclusion:**

InterferenceAnalyzer provides easy access to the powerful and appropriate statistical tools for the multi-locus analysis of genetic data.

## Background

**Figure 1:**
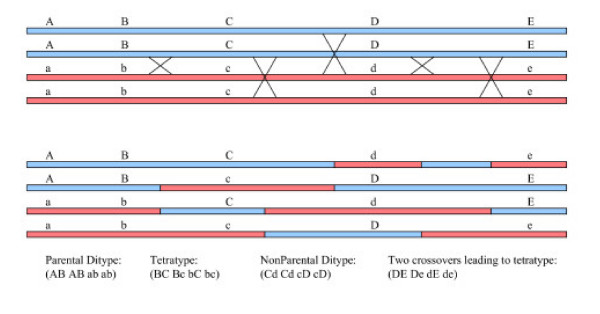
**Geneticist's Data. **The figure demonstrates the three possible tetrad types between pairs of markers, parental ditype, tetratype, and nonparental ditype, with example of how they might arise under specific crossover patterns.

One type of data collected and used by geneticists involves the scoring of markers in a genetic cross of two parents whose markers are known. Such data are used to create genetic maps, associate markers with traits of interest, and to study recombination. For some organisms such as yeast and Arabidopsis, all four products of meiosis can be scored giving rise to tetrad data. Assuming the order of the markers is known or has been inferred, the geneticist can look at adjacent marker patterns and determine if the tetrad has parental ditype, tetratype, or non-parental ditype configuration for the interval. See Figure 1. The simplest explanation for parental ditype is that there are no crossovers in the interval. Similarly, the simplest explanation for tetratype and non-parental ditype patterns are one and two crossovers in the interval, respectively. However, two crossovers in an interval can lead to any of the three possible patterns (parental ditype, tetratype, and non-parental ditype). Under the assumption that the pair of parental strands involved in the crossover is chosen independently for each of the two crossovers (the no chromatid interference assumption), the respective probabilities of these three outcomes are 1/4, 1/2, and 1/4. The no chromatid interference assumption has been supported statistically in most of the experiments where the matter was considered [1,2] and the general formula for the conditional probability of observing any particular tetrad pattern (parental ditype, tetratype, or nonparental ditype) in an interval given the number of crossovers was worked out by Mather in 1935 [3].

The placement of crossovers along the tetrad, however, often does show crossover interference; that is, a crossover discourages another one from occurring nearby. Crossover interference has been observed in many organisms including fruit flies [4-7], yeast [2,8,9], bread mold [4,10], mice [11], humans [12,13], and green plants such as Arabidopsis [14,15]. The only successful statistical model for crossover interference is the counting or Chi-Square model whose mathematical formulation dates back to Payne in 1956 [16] and which was given an elegant formulation in a text of Bailey as *the segmental calculus *in 1961 [17]. If the crossovers were distributed at random, the spacing between them would be exponential, which is equivalent to the scaled Chi-Square distribution 12χ22
 MathType@MTEF@5@5@+=feaafiart1ev1aaatCvAUfKttLearuWrP9MDH5MBPbIqV92AaeXatLxBI9gBaebbnrfifHhDYfgasaacH8akY=wiFfYdH8Gipec8Eeeu0xXdbba9frFj0=OqFfea0dXdd9vqai=hGuQ8kuc9pgc9s8qqaq=dirpe0xb9q8qiLsFr0=vr0=vr0dc8meaabaqaciGacaGaaeqabaqabeGadaaakeaadaWcaaqaaGGaaiab=fdaXaqaaiab=jdaYaaacqWFhpWydaWcaaqaaiab=jdaYaqaaiab=jdaYaaaaaa@322C@. If the spacing between crossovers is the sum of two exponential random variables, then the distribution is the scaled Chi-Square distribution 14χ24
 MathType@MTEF@5@5@+=feaafiart1ev1aaatCvAUfKttLearuWrP9MDH5MBPbIqV92AaeXatLxBI9gBaebbnrfifHhDYfgasaacH8akY=wiFfYdH8Gipec8Eeeu0xXdbba9frFj0=OqFfea0dXdd9vqai=hGuQ8kuc9pgc9s8qqaq=dirpe0xb9q8qiLsFr0=vr0=vr0dc8meaabaqaciGacaGaaeqabaqabeGadaaakeaadaWcaaqaaiabigdaXaqaaiabisda0aaaiiaacqWFhpWydaWcaaqaaiab=jdaYaqaaiab=rda0aaaaaa@3246@. In general, if the spacing between crossovers is the sum of *m *+ 1 exponential random variables, then the distribution is 12(m+1)χ2(m+1)2
 MathType@MTEF@5@5@+=feaafiart1ev1aaatCvAUfKttLearuWrP9MDH5MBPbIqV92AaeXatLxBI9gBaebbnrfifHhDYfgasaacH8akY=wiFfYdH8Gipec8Eeeu0xXdbba9frFj0=OqFfea0dXdd9vqai=hGuQ8kuc9pgc9s8qqaq=dirpe0xb9q8qiLsFr0=vr0=vr0dc8meaabaqaciGacaGaaeqabaqabeGadaaakeaadaWcaaqaaiabigdaXaqaaiabikdaYiabcIcaOiabd2gaTjabgUcaRiabigdaXiabcMcaPaaaiiaacqWFhpWydaqhaaWcbaGae8NmaiJae8hkaGIaemyBa0Maey4kaSIaeGymaeJaeiykaKcabaGae8Nmaidaaaaa@3C26@. The model gained biological credibility when Foss *et al*. [4] proposed that the double strand breaks that initiate recombination events were distributed at random but only every *m *+ 1^st ^one was resolved as a crossover, the intervening ones being resolved with noncrossovers (*i.e.*, simple gene conversions unaccompanied by crossing over.) The number of noncrossovers between pairs of crossovers, *m*, is known as the interference parameter. The counting model has been shown repeatedly to fit genetic data well, both statistically and graphically, and provides a substantially better fit than other statistical models of interference [4,6,7,11,12].

The mathematical details of the use of the segmental calculus for analyzing tetrad data under the counting model and for the extension of the counting model to include an independent subset of crossovers not subject to interference, which provides a better fit to data from Arabidopsis, humans, and yeast, can be found in [7,14]. The basic idea is to use matrices to keep track of the number of noncrossovers to the left (rows) and to the right (columns) of the first and last crossovers in the interval, respectively. The dimensions of the matrices required in the analysis are (*m *+ 1) × (*m *+ 1) where *m *is the interference parameter. The estimate of *m *is chosen to maximize the statistical likelihood function the data.

Calculating the likelihood function involves summing over all possible patterns for the relative positions of the crossovers among the noncrossovers, which is accomplished by the multiplication of matrices that are determined for each interval and each tetrad pattern (parental ditype, tetratype, and nonparental ditype). In the case of the extended model, we also have to sum over all the possibilities for the number of crossovers that are non-interfering. The estimates of *m *(the interference parameter) and *p *(the proportion of crossovers not subject to interference) are chosen jointly to maximize the likelihood function of the data. Since the interference parameters in some organisms can be quite large, the numerical optimization for either model can be quite time-consuming. We save some computational time by using the formula of Perkins [10] for estimating the intermarker distances rather than using maximum likelihood to estimate these values. For most practical applications, the maximum likelihood estimates and the Perkins estimates will be close.

## Implementation

InterferenceAnalyzer is written in Java. The original source code and executable .jar files are available for Windows, Linux, and MacOS. The application is also available as a Windows executable. The source code, executables, sample data sets, and sample results are available at [18].

## Results

We demonstrate how to use the software to analyze a specific dataset, use simulations to give confidence intervals for parameter estimates and assess the significance of the fit of the extended counting model, and discuss the relative speed of our software compared to comparable SAS code.

### Raw data analysis

Genetic data must be in tab-delimited format and stored as a text file. The first two lines specify the parental marker values. The next four lines specify the values for the first tetrad, the four lines after that specify the values for the second tetrad, etc... The file must end with one (and only one) carriage return after the last line of data. Any symbol may be used to specify the parental values for the markers. Common possibilities include the use of the numbers 0 and 1, the use of the symbols + and -, or marker names such as URA and ura. An example of the first 6 lines of two of the common types of codings is provided in Table 1. Note that the first tetrad in the second data set contains entries indicating that the second marker could not be properly scored. Tetrads with entries that match neither parental type or with entries that indicate that a gene conversion occurred at a marker (that is, with 3 or 4 entries for a marker in the tetrad corresponding to the genotype of just one parent) are discarded from the analysis.

**Table 1:** Sample Data. The first 6 lines of two possible formats for the sample data file including the parental marker values and the scoring of the first tetrad. In the second data set, the first tetrad could not be properly scored at the second marker. Any tetrad with any mis-scored marker or any gene conversion at a marker is discarded from the analysis.

	Sample Data					Sample Data			
Parent 1:	-	-	+	-	Parent 1:	ade5,7	URA3	KAN	lys5
Parent 2:	+	+	-	+	Parent 2:	ADE5,7	ura3	kan	LYS5
	+	-	+	-		ADE5,7	xxx	kan	LYS5
First Tetrad	-	-	-	+	First Tetrad	ade5,7	xxx	KAN	lys5
	-	+	+	-		ade5,7	xxx	KAN	lys5
	+	+	-	+		ADE5,7	xxx	kan	LYS5

The file containing the data is uploaded to the software using the "Load File" button on the "Analyze Raw Data" tab of the software. The user may decide to analyze the data only under the original counting model, only under the extended model which allows for a portion of the crossovers to be free from interference, or under both models by checking the appropriate buttons. After the user clicks on the "Analyze" button, a progress bar displays. The progress bar allows the user to know that the program is running but is not a good measure of the time remaining because it is linked to the current value of the interference parameter, *m*, which is allowed to run from 0 to 20. It takes much longer to calculate the likelihood for larger values of *m *than for smaller ones but the program terminates as soon as the peak of the likelihood function has been reached and so often does not reach the larger values allowed for *m*. There is no linear measure available for the time remaining to complete the calculation of the maximum likelihood estimator.

The results are displayed and buttons that allow exporting the results and the intermarker distances to files for use later are displayed. Exporting the intermarker distances is highly recommended in order to be able to use the simulations panel to give confidence intervals for the parameters and assess the significance of the extended model over the original counting model.

**Figure 2:**
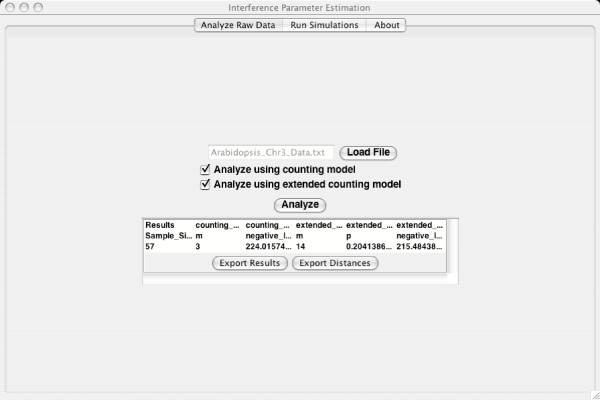
**Analyze Raw Data Panel. **The figure demonstrates the panel for the initial analysis of raw tetrad data. The data file can be uploaded by selecting the "Load File" button. The user can choose the models for the analysis. Then the results are printed on the panel and can be exported to a file. The genetic distances between markers can also be stored for use in the simulations needed to test significance and give confidence intervals.

A picture of this panel of the software is given in Figure 2. The data used for this analysis are the raw tetrad patterns for Arabidopsis chromosome 3 originally analyzed in [14]. Under the original counting model the results show that the estimate of the interference parameter is *m *= 3. Under the extended model, the interference parameter estimate can only increase because the extended model removes a portion of non-interfering crossovers. For the extended model, the results are that the interference parameter is *m *= 14 and the proportion of non-interfering crossovers is *p *= 0.204. The log likelihood ratio, used below to calculate the significance of the increase in fit provided by the extended model, can be calculated as the difference in the log likelihoods provided: 224.0 *- *215.5 = 8.5.

### Use of simulations

The analysis of the original tetrad data discussed in the previous section gives point estimates for the interference parameter, *m*, in the counting model and the interference parameter, *m*, and the proportion of crossovers that are free of interference, *p*, in the extended model. Interval estimates can come from using the asymptotic normality of the maximum likelihood estimators and Fisher's Information function or via simulations. Simulations are more appropriate with small datasets and with large estimates of *m *because the distribution of the maximum likelihood estimators tend not to be close to normally distributed. Also, while we can form the standard likelihood ratio test statistic for determining how much better the extended model fits the data than the original counting model, the null hypothesis that the original counting model is an adequate model for the data is on the boundary of the parameter space (*p *= 0). Thus, the distribution of the usual likelihood ratio test statistic need not be approximately Chi-Square and simulations are needed to accurately assess the significance of the hypothesis that the extended model fits the data better.

#### Confidence intervals for parameter estimates

For the Arabidopsis data, the extended model estimate of the interference parameter was *m *= 14 and the estimate of the proportion of crossovers free from interference was *p *= 0.20. To place confidence intervals around these estimates, we use these parameter estimates from the original data analysis and the estimates of the intermarker genetic distances obtained from the original data using Perkins's formula to simulate new data sets. In each simulated data set, we re-estimate the model parameters *m *and *p*. The variation we see in these estimates reflect our uncertainty in the original parameter estimates. If, for each parameter, we place the simulated values in order and extract the 2.5 and 97.5 percentiles, we obtain the 95% percentile bootstrap confidence interval.

To use the Simulations panel for this purpose, we would load the file containing our intermarker distances, enter the number of tetrads in our original data set (57) in the "Sample Size" textbox, choose *m *= 14 and enter 0.20 in the textbox for *p*, and uncheck the box for analyzing the data using the original counting model since we are not interested in those results.

We give the results for 5 simulations in Table 2. These results demonstrate the limitations of simulations to provide confidence intervals for the interference parameter when that parameter is large and the sample size is small. The largest value for *m *allowed in the program is 20 and the analysis of the data when *m *is large takes an extremely long time. These 5 simulations took approximately 35 minutes on a Dell LATITUDE C840 (Intel Pentium 4 processor) with 1.60 GHz CPU and 1 GB Ram. Also, the maximum likelihood estimate for *m *for the simulated data reached and was truncated at 20 twice in these five simulations. Thus, it is not computationally feasible to place accurate, bounded, confidence intervals around the interference parameter when *m *is large and the sample size is relatively small. Further, the confidence intervals around the proportion of non-interfering crossovers, *p*, is likely to be slightly biased due to the truncation of the interference parameter at *m *= 20.

**Table 2:** Sample Simulation Results. An example of the simulation results

Simulation Results					
Results	counting_...	counting_...	extended_model	extended_model	extended_model

Simulation	*m*	negative...	*m*	*p*	negative_log_likelihood

1			14	0.337	231.22
2			20	0.324	232.92
3			5	0.212	230.34
4			19	0.157	197.78
5			20	0.204	215.51

Since data sets from yeast avoid these problems, we include a set of yeast data generated in the Stahl Lab and analyzed in [2]. The sample size is large (1783 tetrads) and the interference parameter estimates are relatively small (*m *= 3 for the extended model). The estimates for the model parameters for the extended model for the original data set were *m *= 3 and *p *= 0.088. Two hundred simulations of data sets of 1783 tetrads using these parameter estimates for *m *and *p *took approximately eight hours on a Macintosh 1.5 GHz PowerPC G4 laptop computer with 1 GB DDR SDRAM. After exporting the results, opening them in a spreadsheet program, and sorting the data by the interference parameter estimate under the extended model, pulling off the 5^*th *^and 195^*th *^values gives a 95% percentile bootstrap confidence intervals for *m *of [3,4]. Similarly, sorting the data by the proportion of non-interfering crossovers, *p*, and pulling off the 5^*th *^and 195^*th *^values gives a 95% percentile bootstrap confidence interval for *p *of (0.058, 0.135).

#### Assessment of the significance of the fit of the extended model

**Figure 3:**
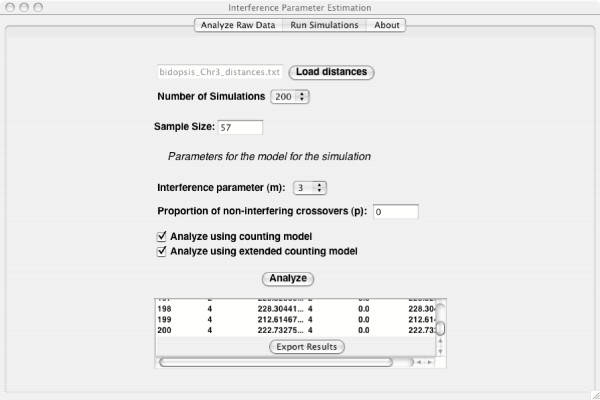
**Run Simulation Panel. **The figure demonstrates the panel for running the simulations needed to assess significance and give confidence intervals. The results are printed at the bottom of the panel. Scrolling to the bottom of the results reveals the button for exporting the results to a file.

For the Arabidopsis data set, we assess the significance of the fit of the extended model by simulating data under the best fitting original counting model (the null hypothesis of the test of whether the extended model fits better or not). Since the estimate of the interference parameter for the original counting model is *m *= 3, the simulations are feasible. We then analyze the simulated data sets under both models. Figure 3 shows the output and the "Export Results" button located at the bottom of the results panel. To determine the significance of the fit of the extended model to these data, the researcher would calculate the difference between the likelihoods of the counting model and extended model for the original data: 224.0 *- *215.5 = 8.5. The researcher would then open the simulation results in a spreadsheet program, calculate a column of such differences (one for every simulation), sort that column, and determine what proportion of the differences are greater than that of the observed data (in this case, 8.5). In our 200 simulations, no difference was greater, so our best estimate of the significance is 0/200. This significance level indicates that the extended model provides a much better fit to the original data than the original counting model does.

### Performance

The intermarker distances for the Arabidopsis data used in our worked example are [0.149, 0.228, 0.132, 0.061, 0.167, 0.219, 0.175]. For 200 simulations with *m *= 3 and *p *= 0 analyzed under both the original counting model and its extension to include a proportion of noninterfering crossovers, InterferenceAnalyzer took approximately 1 hour on a Dell LATITUDE C840 (Intel Pentium 4 processor) with 1.60 GHz CPU and 1 GB Ram whereas the equivalent code in SAS took approximately 16 hours. Thus, the Java program seems to be approximately 16 times faster than similar code in SAS on the same computer.

## Discussion

The development of InferferenceAnalyzer should make the powerful multilocus techniques for assessing interference accessible to geneticists. Future development planned includes allowing for the analysis of spore data where only one product of meiosis is observed, allowing for analysis when positions of the crossovers along a tetrad or spore are known using the algorithms in [12,13], and the inclusion of the ability to simulate data under the mechanical stress model for crossover interference [19]. While the mechanical model does a good job approximating the observed interference patterns in real data, it is not a statistical model and its best fitting parameter values cannot be obtained feasibly from data. Thus our software will not be able to fit the mechanical model to data but only allow the simulation of such data.

## Conclusion

We recognize the need for easy-to-use software in order to make sophisticated and powerful multilocus statistical techniques readily available to all geneticists. InterferenceAnalyzer is our attempt at this goal.

## Availability and requirements

**Project name: **InterferenceAnalyzer

**Project home page: **

**Operating syatems(s): **Platform independent

**Programming language: **Java

**Other requirements: **Java 1.4.1 or higher

**License: **Open source

**Any restrictions to use by non-academics: **None

## Authors' contributions

LV translated the original SAS and Visual Basic code of EAH into Java and programmed the user interface. EAH debugged and refined the Java code, helped design and test the user interface, and wrote this article. Both authors read and approved the final manuscript.
